# Possible monitoring of mesophotic scleractinian corals using an underwater mini-ROV to sample coral eDNA

**DOI:** 10.1098/rsos.221586

**Published:** 2024-02-14

**Authors:** Koki Nishitsuji, Shinichiro Nagahama, Haruhi Narisoko, Kazuo Shimada, Nobuhiro Okada, Yuki Shimizu, Noriyuki Satoh

**Affiliations:** ^1^ Marine Genomics Unit, Okinawa Institute of Science and Technology Graduate University, Onna, Okinawa 904-0495, Japan; ^2^ NTT Communications, Kyushu Office, Naha, Okinawa 900-0025, Japan; ^3^ Docomo Business Solutions, Inc., Kyushu Office, Naha, Okinawa 900-0025, Japan; ^4^ Division of Solution Service, NTT DOCOMO, INC., Minato-ku, Tokyo 107-0052, Japan

**Keywords:** mesophotic scleractinian corals, a possible survey, coral-specific eDNA, underwater mini-ROVs, genera-level detection

## Abstract

Mesophotic coral ecosystems (MCEs) are light-dependent tropical or subtropical communities occurring at depths of 30–150 m. Broader surveys of MCEs are needed to better understand stony corals, the keystone species of coral-reef ecosystems. While MCEs have been studied by professional SCUBA divers and with deep-sea robots, comprehensive surveys of MCEs are required. An eDNA metabarcoding method has recently been used to survey scleractinian corals in shallow reefs. We tested whether MCEs might be more comprehensively surveyed by collecting seawater samples using an underwater mini-remote operated vehicle (mini-ROV). Seawater was collected 1–2 m above reef tops at depths of 20–80 m at 24 sites in six locations around the Zamami Islands (Okinawa, Japan). Water samples were then subjected to coral-specific eDNA amplification. Metabarcoding analyses of amplicons showed that except for one site, coral-specific eDNA from approximately 0.5 l seawater samples was sufficient to identify genera. The proportion of *Acropora* eDNA was higher at shallow reefs and upper ridges of slopes, while the proportion of *Porites* increased at mesophotic sites*.* Although further technical improvements are required, this study suggests that it may be possible to monitor mesophotic corals to the generic level using eDNA collected using mini-ROVs.

## 1. Introduction

Mesophotic coral ecosystems (MCEs) are light-dependent ecosystems occurring at depths of 30–150 m in tropical and subtropical regions, major components of which are corals, sponges and algae [1–4]. Although shallow coral reefs are now in crisis due to climate change and other threats [5], MCEs are probably less threatened by seawater temperature rise, cyclones and pollution [6,7]. MCEs have recently been recognized as unique ecosystems, important in their own right. Growing evidence suggests that MCEs harbour proportionally more geographically endemic species than their shallow-water counterparts and that major biogeographic patterns described for shallow reef organisms may not apply to MCEs [1–4]. However, MCEs still remain largely uncharacterized; therefore, thorough surveys of MCEs are essential to understand their basic biology, including taxonomic composition, depth requirements, geographical distribution, ecology and connectivity.

Hitherto, monitoring of mesophotic corals has depended on direct observation by SCUBA divers who use sophisticated diving equipment and techniques to reach such depths, as well as expert knowledge of coral taxonomy [8,9]. In addition, underwater robots with high-resolution cameras have enhanced our understanding of mesophotic corals [8,9]. Current methods of monitoring MCEs, however, have limited utility for comprehensive surveys, and new methods are needed for this purpose.

An eDNA metabarcoding method has recently been used to monitor scleractinian corals at shallow reefs [10–15]. As of 1 May 2021, mitochondrial genome sequence information for 15 families, 36 genera and 71 species of scleractinians has been deposited in NCBI (https://www.ncbi.nlm.nih.gov/nuccore). Exploiting this information, Shinzato *et al*. developed a set of primers to amplify DNA sequences of mitochondrial 12S-ribosomal-RNA genes (12S rDNA), by which these 36 genera can theoretically be identified from sequence differences [14]. This method was applied to corals along the Onna coast of Okinawa Island and sampling of approximately 1 l of surface seawater was sufficient to identify 23 genera of scleractinian corals [14]. In addition, the method was validated in a broad survey of 63 coral-reef sites (5–15 m in depth) around Okinawa Island, carried out in combination with direct observations by coral-specialist divers [15]. That study confirmed that eDNA metabarcoding can distinguish most directly observed coral genera at more than 90% of monitored locations [15]. Scleractinian-specific eDNA metabarcoding method has two significant advantages compared with methods used for surveying population diversity of fish or other mobile organisms: (i) corals are attached to the substrate and are immobile, and (ii) corals constantly secrete mucus into sea, which provides ample eDNA. Therefore, this method appears to have great potential for thorough surveys of scleractinian corals at shallow reefs [16].

**Figure 1.**
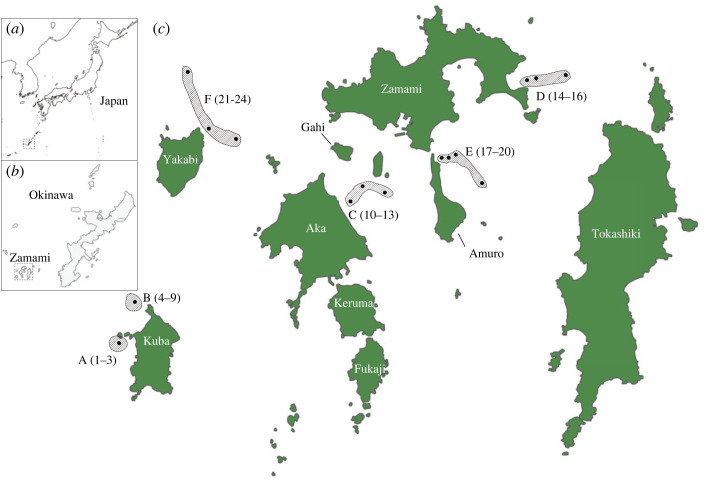
A scleractinian coral eDNA survey at mesophotic sites sampled using an underwater mini-ROV. (*a*) The location of the Okinawa Archipelago in Japan is shown with a dotted square. (*b*) The Zamami Islands are shown with a dotted square. (*c*) Six monitoring locations including 24 sites around the Zamami Islands. A Kuba West, B Kuba Northwest, C Kitahama Beach, D Kerama West, E Shiru East and F Jijigatama (near Yakabi Island). Dots indicate seawater sampling sites with numbers. More detailed information about monitoring dates and locations is available in table 1 and topographic features are found in electronic supplementary material, figure S1.

Recently, the use of underwater mini-remote operated vehicles (mini-ROVs) was suggested for surveying coral reefs [8,9]. Therefore, we employed a mini-ROV to sample eDNA from mesophotic corals at depths of 30–80 m. MCEs in the Okinawa Archipelago of Japan exhibit some of the greatest scleractinian diversity in the world. At least 47 genera belonging to 14 families have been identified [17–19]. Kerama National Park of Japan, approximately 30 km west of Okinawa Island, boasts some of the most transparent water in the Okinawa Archipelago, and is known as ‘Kerama blue’ (figure 1). These coasts therefore afford an excellent opportunity to test this novel sampling technique for coral-specific eDNA to survey MCEs. Here, we examined the efficacy of eDNA metabarcoding for seawater samples of MCEs collected using underwater mini-ROVs.

## 2. Methods

### 2.1. Sampling

This study examined whether scleractinian coral-specific eDNA can be amplified from 500 ml of seawater collected at depths of 20–80 m using a sampler attached to a mini-ROV. Seawater samples were collected 1–2 m above reef tops at 24 sites in six locations around the Zamami Islands on 9–10 March and 23–25 May 2022. Sampling sites differed in depth and geomorphology (sampling sites, depth of sampling, latitude, longitude and sampling dates are summarized in figure 1 and table 1; electronic supplementary material, figure S1). These locations included three types of slope with different features (figure 1; electronic supplementary material, figure S1): Kuba Island West and Kuba Island Northwest possess steep slopes facing the open ocean. Kitahama Beach North, Kerama West and Shill East have gradual slopes in an inland sea, and Jijigatama sites have variable slopes.

**Table 1.** Sampling sites and their names, point numbers, depth of water collection, coordinates and sampling dates.

site	point numbers	depth of water collection	latitude	longitude	sampling date
A. Kuba West	1	SF^a^	26° 17.423′ ^b^	127° 22.865′	9–10 Mar 2022
	2	40 m			
	3	50 m			
B. Kuba Northwest	4	SF	26° 18.219′	127° 23.3′	23 May 2022
	5	9 m			
	6	20 m			
	7	28 m			
	8	40 m			
	9	50 m			
C. Kitahama Beach	10	SF	26° 20.615′	127° 28.625′	24 May 2022
	11	15 m			
	12	28 m	26° 20.838′	127° 28.927′	
	13	42 m	26° 20.804′	127° 29.432′	
D. Kerama West	14	SF	26° 23.519′	127° 33.255′	25 May 2022
	15	20 m	26° 23.537′	127° 33.365′	
	16	40 m	26° 23.55′	127° 34.677′	
E. Shiru East	17	SF	26° 21.689′	127° 31.063′	24 May 2022
	18	20 m	26° 21.694′	127° 31.143′	
	19	41 m	26° 21.708′	127° 31.375′	
	20	50 m	26° 21.113′	127° 32.035′	
F. Jijigatama	21	SF	26° 22.31′	127° 25.013′	24 May 2022
	22	20 m			
	23	41 m	26° 22.145′	127° 25.472′	
	24	80 m	26° 23.482′	127° 24.683′	

^a^Surface.

^b^Blank indicates almost the same as that of surface point.

**Figure 2.**
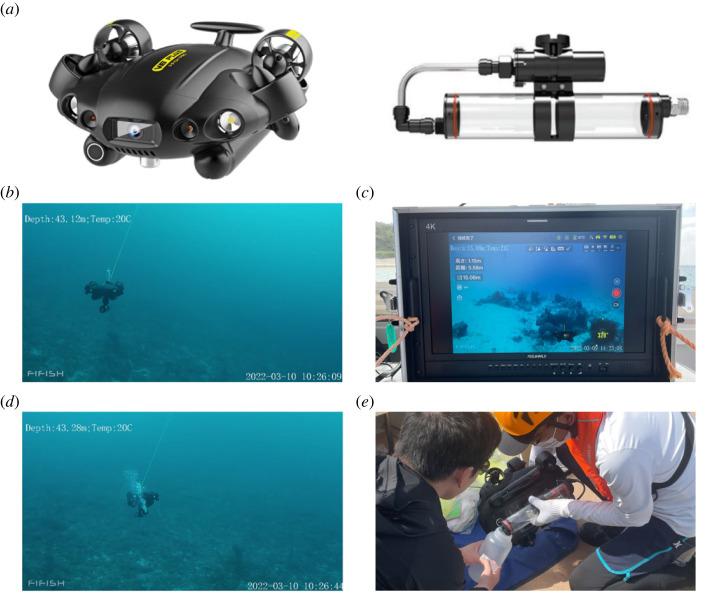
(*a*) A mini-ROV (left) and seawater sampler (right) used in this study to collect 500 ml seawater samples 1–2 m above reef tops. (*b*) View from an observational mini-ROV used to monitor movements of the sampling mini-ROV. See electronic supplementary material, video S1 for control of the mini-ROV. (*c*) Monitoring on the boat for controlling mini-ROV movement. (*d*) Bubbles released from the sampler during seawater collection. See electronic supplementary material, video S2 for seawater collection. (*e*) On-board transfer of a seawater sample from the sampler to a bottle used for filtering.

Mini-ROVs used in this study were FIFISH V6Plus (https://www.qysea.com/jp/products/fifish-v6/) (figure 2*a*) with 150 m cables between the ROVs and their controller. The mini-ROVs had a maximum operating speed of 1.5 m s^−1^. A water sampler (FIFISH model No. QY-WS-500) with a capacity of 500 ml (figure 2*a*) was attached to the bottom of the FIFISH V6Plus. On the control vessel, four NTT-associated mini-ROV technical staff members and three Okinawa Institute of Science and Technology (OIST) scientists worked together. One technical specialist operated the mini-ROVs using controllers (figure 2*b*; electronic supplementary material, video S1). Another specialist provided video-based suggestions about vehicle locations to the specialist (figure 2*c*), and two others were responsible for deploying and retrieving the mini-ROVs. OIST scientists watched the video camera to select locations for water sample collection. The Kerama Islands are a Japanese National Park, from which coral sampling is prohibited; therefore, coral samples could not be collected for comparison with eDNA results.

First, we examined whether underwater mini-ROVs could be controlled in fast currents at depths exceeding 30 m and whether water samples could be collected with the attached sampler. Owing to strong currents, mini-ROVs usually did not remain beneath the ship, and sometimes strayed far from it, at angles greater than 45°. The operators exercised great care so as not to damage corals with the mini-ROVs (figure 2*b*; electronic supplementary material, video S1). To this end, two FIFISH V6Plus, with and without a sampler, were operated simultaneously. The second mini-ROV was used to monitor movements of the sampling mini-ROV (figure 2*b*; electronic supplementary material, video S1). This trial demonstrated that the FIFISH V6Plus could be controlled well enough for collection of seawater samples 1–2 m above the reef without risk to corals. During seawater collection, air in the sampler was replaced by seawater, releasing many air bubbles (figure 2*d*; electronic supplementary material, video S2), so that we could visually confirm water collection at desired sites. The sampler was firmly attached to the ROV (figure 2*b*) and could not be removed between samples, making some cross-contamination of seawater samples inevitable. To reduce this, the sampler was thoroughly rinsed with fresh water after every collection. In addition, triplicate 1 l samples of surface seawater were collected for comparative examination of shallow-water eDNA.

### 2.2. eDNA extraction and sequencing

On the boat, seawater collected by the sampler was transferred to 1 l bottles (figure 2*e*) and filtered promptly through 0.45 μm Sterivex filters (Merck) using a peristaltic pump. One millilitre of RNAlater (Qiagen) was added to the filters to prevent DNA degradation [14], and samples were maintained at 4°C before transfer to a −20°C freezer in the laboratory. eDNA in Sterivex filters was extracted following instructions in the Environmental DNA Sampling and Experiment Manual v. 2.1 [20]. Extracted eDNA samples were PCR-amplified using primers, Scle_12S_Fw (5′-CCAGCMGACGCGGTRANACTTA-3′) and Scle_12S_Rv (5′-AAWTTGACGACGGCCATGC-3′), for mitochondrial 12S rRNA genes of scleractinian corals, as described in Shinzato *et al*. [14]. By adding newly registered sequences, these primers were designed to identify 45 scleractinian coral genera [15], and the present study identified 34 genera, as described in the Results. PCR amplification was carried out with Tks Gflex DNA Polymerase (Takara) under cycling conditions of 1 min at 94°C, followed by 35 cycles of 10 s at 98°C, 15 s at 60°C and 30 s at 68°C, with an extension of 5 min at 68°C in the final cycle. PCR products were extracted and cleaned with a FastGene Gel/PCR Extraction Kit (NIPPON Genetics Co., Ltd). Amplicon sequencing libraries of cleaned PCR products were prepared using a KAPA Hyper Prep Kit (NIPPON Genetics) without fragmentation. Libraries were multiplexed and 300 bp paired-end reads were sequenced on a MiSeq platform (Illumina) using a MiSeq Reagent kit v3 (600 cycles). The number of sequence reads, total base-pair length, and average and maximum length of reads of each sample are shown in electronic supplementary material, table S1.

### 2.3. Bioinformatic analysis

Bioinformatic analyses of eDNA sequence data were carried out essentially as described previously [14,15]. First, low-quality bases (Phred quality score less than 20) and Illumina sequencing adapters were removed [21]. Sequences greater than or equal to 200 bp were retained. Merging of paired reads, unique sequence identification, chimera removal and denoising (error-correction) were performed using USEARCH, v. 11.0.667 [22]. Then, denoised (error-corrected) operational taxonomic units, called zero-radius operational taxonomic units (ZOTUs), were prepared for each sample. ZOTU sequences from all samples were concatenated and clustered using CD-HIT-EST v. 4.6 with 100% nucleotide identity [23]. Clustered, unique ZOTU sequences were used for a database for mapping. Merged sequences from each sample were mapped to clustered ZOTUs and numbers of mapped sequences for each ZOTU were counted using the USEARCH ‘otutab’ command with 99% identity (-id 0.99).

ZOTUs originating from scleractinians were selected based on criteria described in our previous study [15]. After selecting scleractinian ZOTUs, mapped reads from the same genera were combined. We removed scleractinian genera restricted to the Atlantic Ocean and only genera with more than 0.01% of the total number of mapped reads in a given sample were considered. Percentages of coral genera detected in each sample were shown with the heatmap command in R v. 4.1.2 [24].

This eDNA method can determine the presence or the absence of corals at the generic level and their proportions at a given site. However, this method cannot validate abundance estimates of coral genera.

## 3. Results

**Figure 3.**
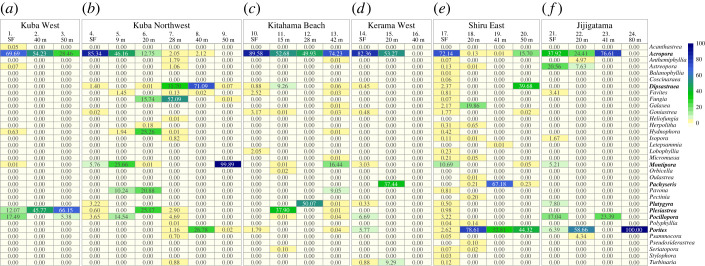
Percentages of sequence reads mapped to coral genera in each eDNA sample. Percentages are coloured in the heatmap. (*a*) Kuba West, (*b*) Kuba Northwest, (*c*) Kitahama Beach, (*d*) Kerama West, (*e*) Shiru East, and (*f*) Jijigatama (near Yakabi Island). Monitoring sites are numbered, and depths of sites are shown. SF, surface seawater of shallow coral reefs.

**Figure 4.**
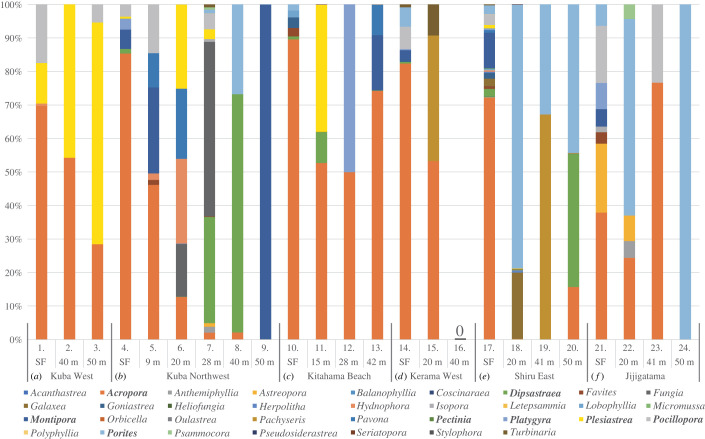
Bar graph showing the distribution and approximate proportions of scleractinian corals at each monitoring site at the Kerama Islands. Names of scleractinian coral genera are shown in different colours at the bottom. Numbers 1–24 indicate eDNA sampling sites with approximate depths in metres. SF means surface seawater.

We amplified coral-specific eDNA from all sites, except one (figure 3*d*) (figures 3 and 4; electronic supplementary material, table S1), confirming the feasibility of eDNA metabarcoding using seawater collected by mini-ROVs (figure 3). The site at which we failed to amplify scleractinian-specific eDNA was site 16, at a depth of 40 m (Kerama West) (figure 3*d*). However, eDNA was amplified from seawater collected at the surface and at 20 m depth at the same location (figure 3*d*). The mini-ROV's video showed that this site was covered mostly by soft corals and/or seaweed, with very few scleractinian corals (electronic supplementary material, figure S2A). Therefore, it is likely that site 16 samples contained little or no scleractinian coral eDNA.

This study sought to determine whether this sampling method could detect and identify scleractinian corals by their DNA sequence differences. Thirty-four genera were scored in the ZOTU analyses, including *Acropora, Dipsastraea, Galaxea, Isopora, Montipora*, *Pachyseris, Pocillopora* and *Porites* (figure 3; electronic supplementary material, table S2). *Millepora* is also common in this area (electronic supplementary material, figure S3), but was not detected by this method, since it is not a scleractinian and the PCR primers could not recognize its DNA. Therefore, seawater collection with a mini-ROV in combination with eDNA metabarcoding appears to be a viable way to survey MCEs.

The presence and absence of scleractinian coral genera shown by this method demonstrated that reefs around the Kerama Islands exhibited different scleractinian compositions in relation to location and depth (https://www.biodic.go.jp/moni1000/manual/spot-check_ver5.pdf). *Acropora, Dipsastraea, Montipora, Pachyseris, Pocillopora* and *Porites* were commonly detected at Zamami Island reefs, based on higher ZOTU scores at many sites (figure 3). In particular, *Acropora* had the highest ratios at 11 sites, indicating that it is a common genus at Zamami Island reefs (figures 3 and 4; electronic supplementary material, table S2). At the same time, most sites featured two to six genera, whereas two sites, 9 (50 m depth at Kuba Northwest) and 24 (80 m depth at Jijigatama), indicated only one genus, the former being *Montipora* and the latter *Porites* (figures 3 and 4; electronic supplementary material, table S2).

In regard to depth, *Acropora* was readily detected at shallow reefs (less than or equal to 15 m), whereas other genera were more frequently found at deeper reefs (greater than 20 m) (figures 3 and 4). At five locations other than Jijigatama, *Acropora* comprised more than 65% of coral genera detected in surface seawater eDNA (figures 3 and 4). At greater depths, the percentage of *Acropora* decreased, and ratios of other genera increased. For example, *Plesiastrea* was commonly identified at depths of 40 and 50 m at Kuba Island West (figures 3*a* and 4*a*) and *Dipsastraea* and *Montipora* were frequently identified at depths of 40 and 50 m at Kuba Island Northwest (figures 3*b* and 4*b*). At Shiru East, *Pachyseris* was found at 41 m and *Dipsastraea* and *Porites* at 50 m (figures 3*e* and 4*e*).

The decrease in proportions of *Acropora* became evident at a depth of approximately 20 m. For instance, at Kuba Island Northwest, approximately 90% of ZOTUs in surface seawater were from *Acropora*, but *Acropora* eDNA decreased to 13% at 20 m (figures 3*b* and 4*b*). Instead, *Hydnophora, Pavona* and *Plesiastrea* accounted for approximately 20–25% at this depth (figures 3*b* and 4*b*). Similarly, at Shiru East, *Acropora* ZOTUs that comprised more than 70% of the surface sample disappeared almost entirely at 20 m, whereas *Porites* accounted for 79% of coral eDNA at that depth (figures 3*e* and 4*e*). One exception, however, occurred at Kitahama Beach South, at which a high ratio (approx. 90%) of *Acropora* in shallow water decreased to approximately 50% at 28 m, but accounted for 75% at 42 m (figures 3*c* and 4*c*).

A depth-dependent linear change in coral generic composition was probably observed at Kuba Island West (figures 3*a* and 4*a*). In surface seawater, the most heavily documented genus was *Acropora* (approx. 67% of all ZOTUs). However, the ratio was 54% at 40 m and 28% at 50 m (figure 3*a*; compare sites 1–3 in figure 4*a*). In contrast, proportions of *Plesiastrea* ZOTUs in surface seawater, 40 m and 50 m were 15%, 46% and 66%, respectively (figures 3*a* and 4*a*).

## 4. Discussion

It has become apparent that MCEs are unique ecosystems, since they harbour proportionally more geographically endemic species than their shallow-water counterparts [1–4]. However, MCEs remain largely uncharacterized, awaiting thorough surveys. More comprehensive surveys of MCEs will facilitate understanding of their basic biology, including taxonomic composition, depth requirements, geographical distribution, ecology and connectivity. Until now, MCEs have been surveyed predominantly by direct observations by SCUBA divers and underwater robots with high-resolution cameras [8,9]. This study sought to develop another way to survey MCEs, using the simpler, more convenient method of eDNA metabarcoding of samples collected using a mini-MOV. This report is probably the first survey of MCEs using this method.

eDNA metabarcoding analyses have recently been introduced to monitor corals at shallow reefs [10–15]. As mentioned in the Introduction, the eDNA metabarcoding method is more robust for surveying scleractinians than for surveying fish or other mobile organisms. This is because (i) corals are attached to the substrate, so they are immobile, and (ii) corals constantly secrete mucus into the sea, which provides ample eDNA. However, other concerns have recently been raised about technical problems with eDNA metabarcoding methods when used for fish or microbiota [25,26]. Scleractinian-specific eDNA metabarcoding will undoubtedly require some methodological improvements; nonetheless, its potential is readily apparent.

In addition, using mini-ROVs to collect eDNA for surveying mesophotic coral ecosystems will require technical refinements and necessitates cautious interpretation of results in the interim [15,16]. The V6 mini-ROV can carry only one 500 ml seawater sampler; however, it is preferable to collect duplicate or triplicate eDNA samples at each site [15]. Therefore, development of mini-ROVs with two or three samplers is essential for further monitoring using this methodology. Another problem raised by this study is cross-contamination of water samples, since the sampler is firmly attached to the mini-ROV and cannot be removed. Therefore, small amounts of seawater remaining on the inner wall of the sampler may have caused slight contamination of subsequent samples, even though we washed the sampler with fresh water between samples. A more effective method to prevent cross-contamination, such as detachable samplers, should be considered for future studies. Furthermore, limitations on electronic battery power should also be resolved. This study sought to collect as many seawater samples as possible; therefore, most electric power was needed for operation of the mini-ROV, so we did not use lighting to observe corals. Therefore, most photos of collection sites were of insufficient quality to identify coral genera clearly (electronic supplementary material, figure S2B–D). It remains uncertain whether the coral genus observed at site 9 (50 m depth) of Kuba Northwest is *Montipora,* and whether *Porites* is present at site 24 (90 m depth) at Jijigatama*,* although both seem likely. These technical issues limit the amount that can presently be said about community composition at these depths.

Recent studies showed that Japanese MCEs exhibit some of the highest scleractinian diversity in the world, making them challenging to monitor [17–19]. *Seriatopora hystrix* is one of the most common species [27]; however, the present study failed to detect *S. hystrix* in Zamami mesophotic ecosystems because the mitochondrial genome of *Seriatopora* has not yet been sequenced, preventing its inclusion in scleractinian-specific eDNA metabarcoding analyses. As mentioned above, among approximately 1300 scleractinians species, comprising 236 genera and 25 families, mitochondrial genome sequence information for only 71 species, 36 genera, and 15 families has been deposited in NCBI (https://www.ncbi.nlm.nih.gov/nuccore). Therefore, further sequencing of mitochondrial genomes of scleractinians to be used for ZOTU analysis is essential for eDNA metabarcoding studies of corals, including species in MCEs. In conclusion, although further technical improvements are required, this study suggests that monitoring of mesophotic corals using eDNA collected by mini-ROVs should be feasible at the generic level.

## Data Availability

Data are available from the Dryad Digital Repository: https://doi.org/10.5061/dryad.kprr4xh82 [28]. Supplementary material is available online [29].
